# Differential Evolutionary Fate of an Ancestral Primate Endogenous Retrovirus Envelope Gene, the EnvV *Syncytin*, Captured for a Function in Placentation

**DOI:** 10.1371/journal.pgen.1003400

**Published:** 2013-03-28

**Authors:** Cécile Esnault, Guillaume Cornelis, Odile Heidmann, Thierry Heidmann

**Affiliations:** 1 Unité des Rétrovirus Endogènes et Éléments Rétroïdes des Eucaryotes Supérieurs, Unité Mixte de Recherche 8122, Centre National de la Recherche Scientifique, Institut Gustave Roussy, Villejuif, France; 2 Université Paris-Sud, Orsay, France; 3 Université Paris Diderot, Paris Sorbonne Cité, Paris, France; University of Utah, United States of America

## Abstract

*Syncytins* are envelope genes of retroviral origin that have been co-opted for a role in placentation. They promote cell–cell fusion and are involved in the formation of a syncytium layer—the syncytiotrophoblast—at the materno-fetal interface. They were captured independently in eutherian mammals, and knockout mice demonstrated that they are absolutely required for placenta formation and embryo survival. Here we provide evidence that these “necessary” genes acquired “by chance” have a definite lifetime with diverse fates depending on the animal lineage, being both gained and lost in the course of evolution. Analysis of a retroviral envelope gene, the *envV* gene, present in primate genomes and belonging to the endogenous retrovirus type V (ERV-V) provirus, shows that this captured gene, which entered the primate lineage >45 million years ago, behaves as a *syncytin* in Old World monkeys, but lost its canonical fusogenic activity in other primate lineages, including humans. In the Old World monkeys, we show—by *in situ* analyses and *ex vivo* assays—that *envV* is both specifically expressed at the level of the placental syncytiotrophoblast and fusogenic, and that it further displays signs of purifying selection based on analysis of non-synonymous to synonymous substitution rates. We further show that purifying selection still operates in the primate lineages where the gene is no longer fusogenic, indicating that degeneracy of this ancestral *syncytin* is a slow, lineage-dependent, and multi-step process, in which the fusogenic activity would be the first canonical property of this retroviral envelope gene to be lost.

## Author Summary

*Syncytins* are “new” genes encoding the envelope protein of captured endogenous retroviral elements. Their unambiguous status of “cellular gene” was recently demonstrated by knocking them out in genetically modified mice, showing their absolute requirement for placenta formation and embryo survival, via formation by cell–cell fusion of the feto-maternal syncytium interface. These genes are remarkable, as they are “necessary” for a basic function in placental mammals and yet they were acquired “by chance” on multiple occasions and independently in diverse mammalian species. We proposed that *syncytins* have been pivotal for the emergence of animals with a placenta from those laying eggs via the capture of a founding retroviral *env* gene, then subsequently replaced in the diverse mammalian lineages upon successive and independent germline infections by new retroviruses and co-optation of their *env* gene, each new gene providing its host with a positive selective advantage. This hypothesis would account for the diversity of the captured *syncytins* that can be currently found, concomitant with the diversity of placental architectures. A consequence of this paradigm is that evidence for “decaying *syncytins*” in eutherian mammals should exist, and this is precisely what we sought—and found—in this study.

## Introduction

*Syncytins* are genes of retroviral origin that have been co-opted by their host for a function related to placentation. They correspond to the *envelope* (*env*) gene of ancestral retroviruses that entered the germline of evolutionarily distant animals and were endogenized (reviewed in [1], [2]). Two such genes have already been identified in simians, namely *syncytin-1* [3]–[7] and *-2* [8], [9], as well as two distinct, unrelated ones in muroids, *syncytin-A* and *–B* [10], one in leporids, *syncytin-Ory1* [11], one in carnivores, *syncytin-Car1* [12], and more recently one in ruminants, *syncytin-Rum1* [13]. Their canonical characteristic features -allowing them to be named “syncytins”- comprise i) placenta-specific expression, ii) cell-cell fusion activity, and iii) conservation in evolution of mammalian species for extended periods of time [e.g. >10 million years (My)]. Syncytin proteins are expected to participate in the formation of the placental syncytiotrophoblast at the maternal-fetal interface, via fusion of the mononucleated cytotrophoblasts [3], [14]–[18]. Some of them additionally possess an immunosuppressive activity, as classically observed for infectious retroviral envelope glycoproteins, which may be involved in maternal-fetal tolerance [19]. The direct involvement of *syncytins* in placentation has been recently demonstrated unambiguously through the generation of knockout mice for *syncytin-A* and *–B* [20], [21], whose embryonic placenta displayed defects in cell-cell fusion, resulting in decreased maternal-fetal exchange and impaired embryo survival.

A remarkable feature of *syncytins* is that these genes, which have been acquired “by chance”, repeatedly and independently in the course of evolution, are “necessary” for a basic function in placental mammals (reviewed in [2]). It has therefore been proposed that syncytins might be present in all placental mammals, and that the capture of a founding syncytin by an oviparous ancestor has been pivotal for the emergence of placentation -approximately 150 My ago. This founding syncytin would then have been subsequently replaced in the diverse emerging mammalian lineages, upon successive and independent germline infections by new retroviruses and co-optation of their *env* gene, each new gene providing its host with a positive selective advantage. This would account for the diversity in the nature and age of the captured *syncytins* that can be presently identified, concomitant with the diversity of placental architectures [2]. A consequence of this evolutive scenario is that evidence should exist for “decaying *syncytins*”, which could possibly be unraveled in eutherian mammals.

In fact, screening of the human genome for envelope protein-coding sequences [22]–[24] led to the identification, in addition to *syncytin-1* and *syncytin-2*, of three *env* genes that share some but not all of the characteristic features of *syncytins*: the *envR*, *envV* and *envPb* genes. The *envR* gene is strongly expressed in the placenta [22], [25] but lacks fusion activity due to a stop codon before the membrane-anchoring domain of the protein, that most probably arose very early in primate evolution being already present in Old World monkeys [26]. The *envV* gene is specifically expressed in the placenta, but its fusogenicity could not be demonstrated, either due to an intrinsic defect, or to the lack of its cognate receptor on the panel of cells used for the *ex vivo* cell-cell fusion assay [24]. Yet, *envV* can be found in all simians, with the orthologous copy displaying a complete open reading frame (ORF) suggesting that it has been subject to purifying selection (although its function was not investigated) [27]. Finally, *envPb* in humans was demonstrated to be fusogenic, and orthologous copies can be found in most simians, but it is only poorly expressed and in a non-specific manner in all the human tissues tested, including the placenta [24], [28]. Altogether, close examination of the status of these *env* genes shows that, presently, they cannot be formally considered as *syncytins*, but still possess some characteristic features suggesting that they could be the remnants of ancestrally co-opted *syncytins* that are progressively losing their function in some primates. This could be a consequence of the incorporation into the genome of the latter of “new” *syncytin* genes –such as *syncytin-1* and *syncytin-2*- which might have functionally replaced them in the course of primate evolution, for *syncytin-2* about 60 My ago (Mya), after the divergence between prosimians and simians, and for *syncytin-1* about 45 Mya, after the divergence between New and Old World monkeys [29]. In fact, the natural history and the time course of these processes might be different depending on the *env* gene. For instance, the intrinsic lack of fusogenicity of *envR* due to a stop codon upstream of the membrane-anchoring domain sequence in all simians, the complete loss of the gene in the gorilla [26], and finally the occurrence of a high polymorphism in this gene among the human population, with 1% of the population being homozygous for a premature second stop codon subsequently acquired in the N-terminal domain of the protein [30], strongly suggest an early loss of function in placentation. In humans, *envV* has no demonstrable fusogenic activity, but this property has not been assayed for the orthologous genes found in other simians, where it could be detected. Here, we cloned the *envV* genes from a series of primates where they have been shown to be present over a 45 My period of time, from New World monkeys to humans, and assayed the cloned genes for their fusogenic activity. A scheme for the “life cycle” of *syncytin* genes in evolution, with recurrent new incorporation into and progressive decay within the genomes of their hosts can be proposed to account for the observed status of these genes in placental mammals.

## Results

### Cloning *envV* from simians

**Figure 1:**
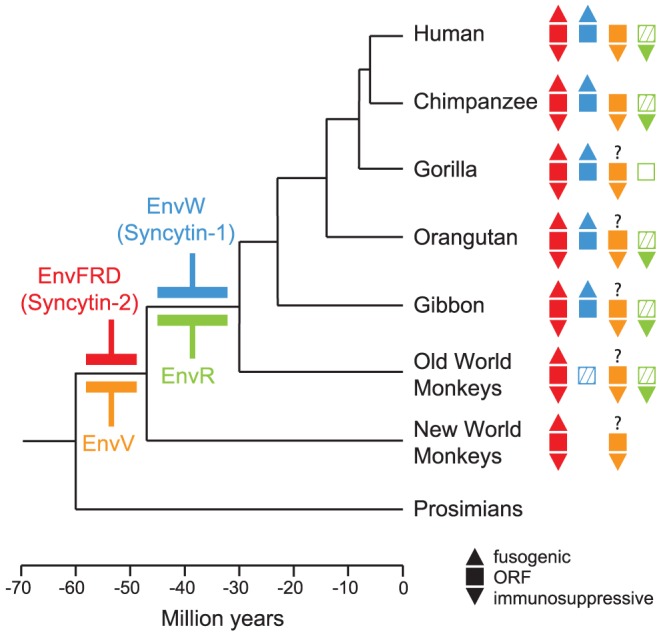
Phylogenetic tree of primates, and status of the captured endogenous retrovirus envelope genes with placental expression. The date of insertion of the indicated retroviral envelope genes into the genome of primate ancestors is indicated for the *syncytin* genes (*i.e.* the EnvFRD- and EnvW-encoding genes, in red [8] and blue [6], respectively, and for the EnvV- and EnvR-encoding genes, in orange [27] and green [26], respectively). Their presence in the various primate lineages is indicated with filled squares when the gene still possesses a full-length ORF, with hatched squares when the coding sequence is prematurely interrupted, and an empty square when the gene is no longer present. In addition, fusogenic activities (as determined by ex vivo cell-cell fusion assays in ref [5], [8]) are schematized with an upper triangle when present, and immunosuppressive activities (as assayed in ref [19]) with lower triangles. Branch length is proportional to time (in million years).

**Figure 2:**
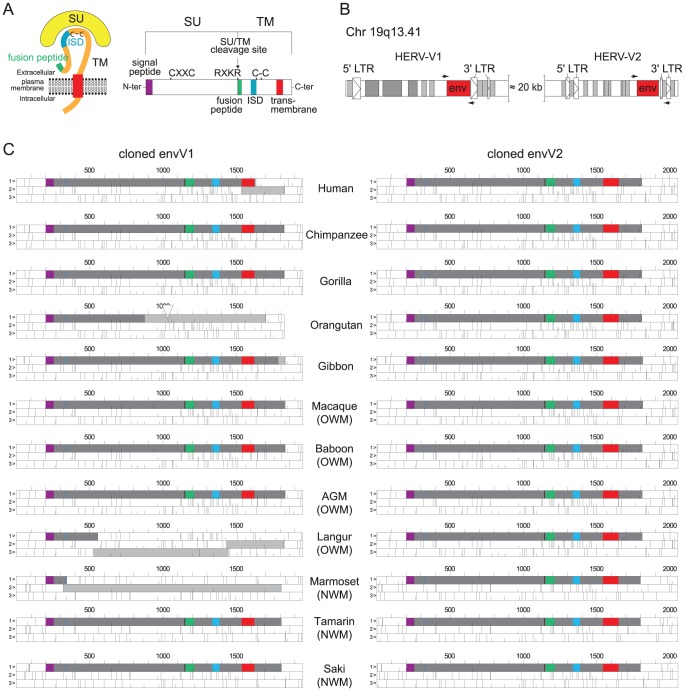
Structure of a canonical retroviral envelope protein and characterization of the *envV1* and *envV2* genes in primates. (A) Schematic representation of a retroviral Env protein, delineating the surface (SU) and transmembrane (TM) subunits. The furin cleavage site (consensus: R/K-X-R/K-R) between the two subunits, the C-X-X-C motif involved in SU-TM interaction, the hydrophobic signal peptide (purple), the fusion peptide (green), the transmembrane domain (red), and the putative immunosuppressive domain (ISD) (blue) along with the conserved C-C motif are indicated. (B) Genomic organization of the HERV-V1 and HERV-V2 proviruses. The retroviral *env* ORF (red open box) and long terminal repeats (LTRs; arrowed open boxes), and the Alu (light gray boxes) and MER50 (dark gray boxes) retroelements are indicated. Positions of the primers designed to amplify the *env* coding sequences are indicated. (C) ORF map of the cloned *envV* genes. The dark gray boxes delineate the envelope coding sequences and the light gray boxes represent the ORFs still present downstream of stop codons or frameshifts. The deletion in the orangutan *envV1* gene is depicted by an open triangle.

Previous studies have shown that ERV-V is a very ancient endogenous retrovirus [24], which entered the primate genome after the simian and prosimian divergence but before the separation of New World and Old World monkeys, more than 45 Mya (Figure 1) [27]. It was also shown that the unique human *envV* gene -at chromosomal position 19q13.41- is in fact part of a post-integrative provirus duplication, with the *envV1* (C-terminally truncated) and *envV2* (full-length) gene sequences −20 kb apart- displaying >94% nucleotide identity (Figure 2A, 2B) and frequent events of gene conversion [27]. Using the human *env* genes as a query, we screened orthologous *env* genes in the available primate genome libraries (http://genome.ucsc.edu and http://www.ensembl.org), in order to design specific PCR primers flanking either the *envV1* or the *envV2* gene. Using appropriate primer pairs, both genes were PCR-amplified and cloned into a CMV-driven expression vector to assay their functional activity. Amplified *envV1* and *envV2* sequences could be recovered from a wide range of simian species, including hominoids (human, chimpanzee, gorilla, orangutan and gibbon), Old World monkeys (macaque, baboon, African green monkey and langur) and New World monkeys (marmoset, cotton top tamarin and white-faced saki), consistent with their previously documented occurrence in the simian lineage (they were not found in prosimians) [27]. For each amplified fragment, several cloned genes were sequenced to recover only those devoid of PCR-induced mutations. The reference sequences (sequences deposited in GenBank with accession numbers KC010496-KC010519) were determined by sequencing the whole PCR product before cloning, and were confirmed -when available- by the corresponding sequences found in genome databases.

As illustrated in Figure 2C, all primate *envV2* genes among the 12 species tested display a full-length ORF (with high sequence conservation, see below), whereas the *envV1* genes were severely altered, with evidence for frameshift mutations (human, langur and common marmoset), stop codons (gibbon and orangutan) and deletion (orangutan) (Figure 2C). Of note, the other primate *envV1* genes, namely from chimpanzee, gorilla, macaque, baboon, African green monkey, cotton top tamarin and white-faced saki, have a full-length ORF (still with significant sequence conservation, *i.e.* 84 to 99% nucleotide identity, most probably due to previously described gene conversion events with *envV2* [27]), and all were therefore also cloned for further studies.

### Functional assay: Cell–cell fusion

**Figure 3:**
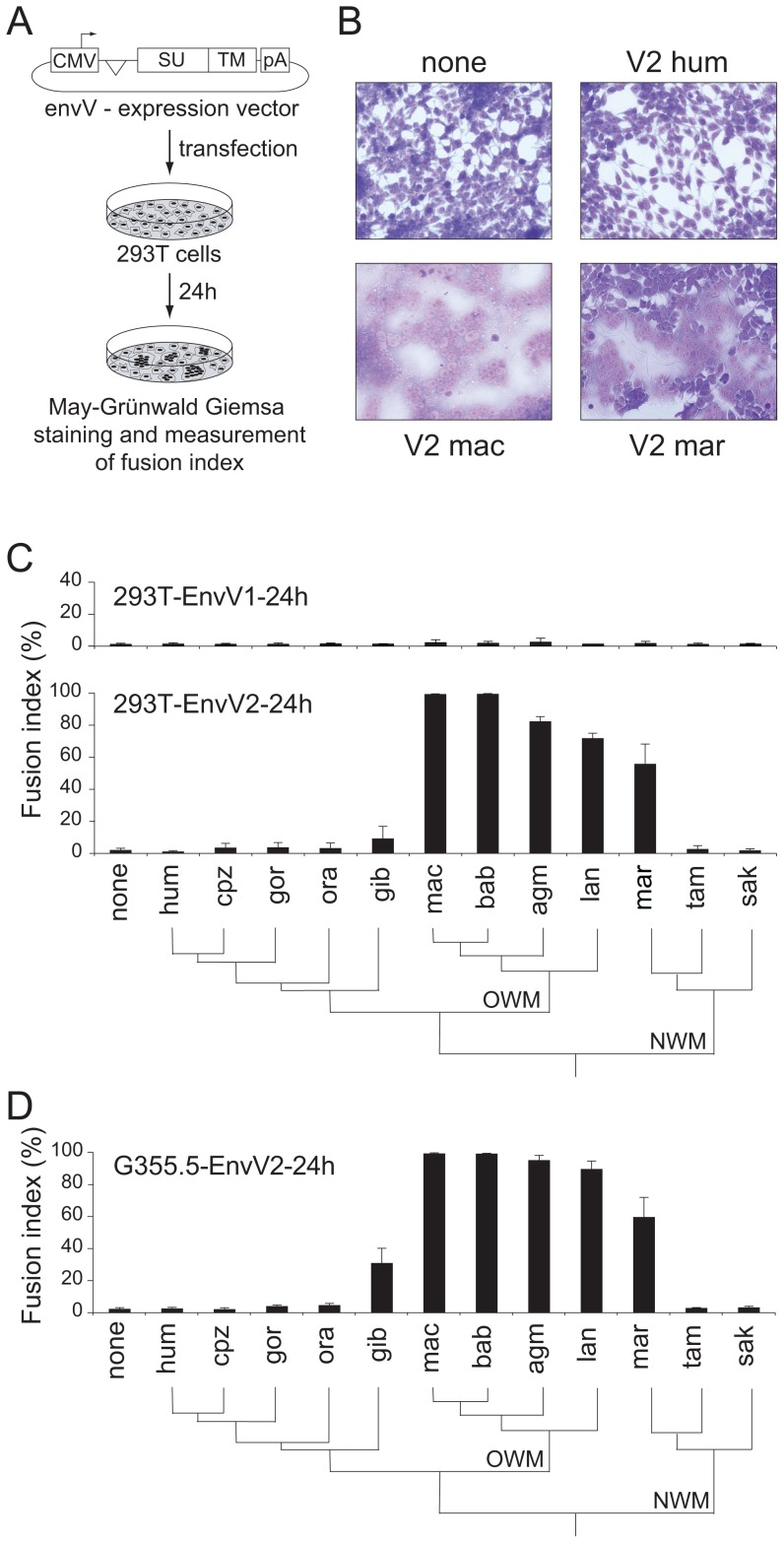
Cell–cell fusion assay for the primate EnvV proteins. (A) Env-expressing vectors and rationale of the assay. Each of the 24 cloned *envV* genes was introduced into the phCMV expression vector, between the beta-globin intron and polyadenylation (pA) sequences. Cells were transfected and stained with May-Grünwald-Giemsa 24 h after transfection. The fusion index is defined as [(*N*-*S*)/*T*]×100, where *N* is the number of nuclei in the syncytia, *S* is the number of syncytia, and *T* is the total number of nuclei counted. (B) 293T cells transfected with expression vectors for no protein (none), and for the human, macaque and marmoset EnvV2 (V2 hum, V2 mac and V2 mar, respectively), displaying large multinucleated syncytia 24 h later, only upon transfection with the latter two. (C) Histogram showing the fusion index of the indicated series of primate *envV1* (upper) and *envV2* (lower) genes in 293T cells transfected with the corresponding expression vectors. The primate phylogeny is illustrated below, with the Old World monkeys (OWM) and New World monkeys (NWM) indicated. None, no protein; hum, human; cpz, chimpanzee; gor, gorilla; gib, gibbon; mac, macaque; bab, baboon; agm, African green monkey; lan, langur; mar, marmoset; tam, tamarin; sak, saki. (D) Same as (C) but with feline G355.5 cells transfected with the *envV2* expression vectors.

**Figure 4:**
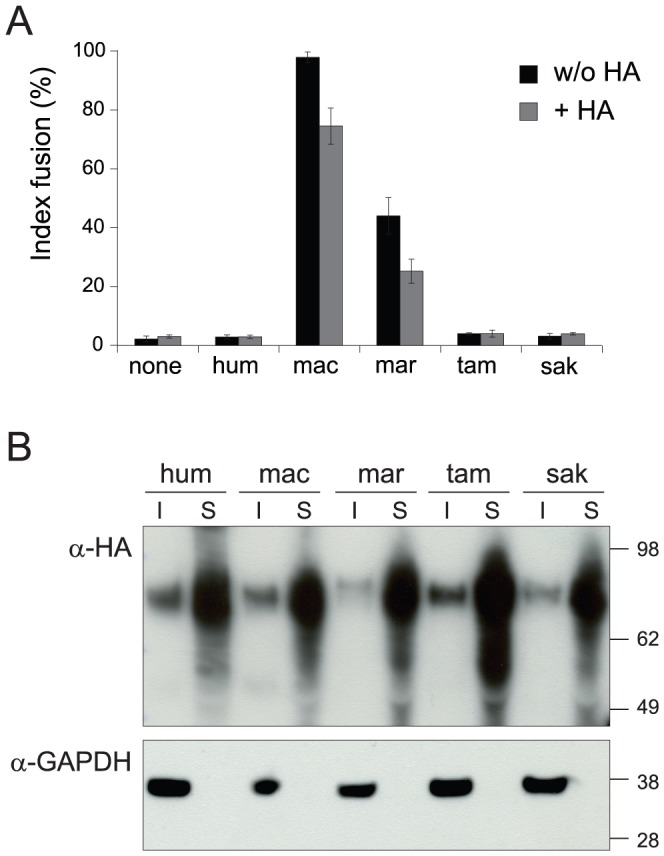
EnvV2 protein expression and cell surface localization. (A) Cell-cell fusion assay for hemagglutinin (HA)-tagged EnvV2 proteins in G355.5 cells. Transient transfection of feline cells was assayed with a control plasmid (none) or plasmids expressing the EnvV2 protein, tagged (or not) with the HA epitope. May-Grünwald-Giemsa staining was carried out 24 h posttransfection and fusion index measured as in Figure 3. (B) Cell-surface expression of EnvV2 proteins. A23 cells were transfected with the indicated EnvV2-HA expression vectors. The cell surface-expressed proteins were biotinylated for further purification (see Methods). Detection of the EnvV2 proteins was performed by Western blotting of the two cellular fractions obtained –*i.e.* the intracellular cell lysates (I) and the cell surface biotinylated proteins (S)- with a monoclonal anti-HA antibody (upper). As a control for the fractioning, the blot was hybridized with a monoclonal antibody against the GAPDH soluble cellular protein (lower).

The main expected function for captured retroviral *env* genes with specific expression in the placenta and involvement in syncytiotrophoblast formation is cell-cell fusion. This was assayed as previously described [8] by transient transfection of cell lines in culture with the above *env*-expression vectors, and follow-up of syncytium formation 1–2 days post-transfection (Figure 3A, 3B). As illustrated in Figure 3B, 3C and in agreement with previously published results [24], the human *envV1* and *envV2* genes were found to have very limited –if any- fusogenic activity. However, and rather surprisingly, Figure 3C also shows that all four tested *envV2* genes from Old World monkeys are highly fusogenic, as well as that from marmoset –but not that from the two other New World monkeys, *i.e.* tamarin and saki. It can also be observed that the *envV2* genes from gibbon, which is part of the hominoids, display a weak, intermediate fusogenicity, and that *envV1* from all simians is fusion-negative (Figure 3C). Of note, the data in Figure 3B, 3C were obtained with human 293T cells, *i.e.* with cells where the receptor for EnvV –still to be identified- should in principle be best fitted to the human EnvV protein. To eliminate any possible artifact due to unexpected species-dependent receptor properties that would discriminate between the Old World monkey –and the New World monkey marmoset- and the other primate EnvV2 proteins, we carried out the same assay with the evolutionarily “remote” G355.5 cat cells (from the superorder Laurasiatheria, which diverged from the superorder Euarchontoglires to which belong primates more than 100 Mya): as illustrated in Figure 3D, the same fusion profile was obtained (and similarly with other cell lines from human, simian and rodent), thus strongly suggesting that the observed differences are intrinsic differences in fusogenic activity of the EnvV2 proteins. To analyze further the molecular basis of the observed differences, we carried out a series of control experiments. First, we checked that representative cloned *envV2* genes that are fusion-negative indeed directed the expression of a membrane-associated protein, similarly to fusion-positive genes used as controls. To do so, we tagged the Env proteins from the 3 New World monkey genes as well as from the human and macaque genes, with the hemagglutinin (HA) epitope. We placed it at the C-terminus of the protein, where the HA-tag is less likely to alter protein folding and function. Indeed, as illustrated in Figure 4A, the HA-tag had only a minimal effect, if any, with the macaque and marmoset EnvV2s being still fusogenic, and the human as well as the two tamarin and saki New World monkey EnvV2s still fusion-negative. Expression of the proteins at the level of the plasma membrane was then investigated by Western blotting, after biotinylation and subsequent streptavidin isolation of the biotinylated cell surface proteins. As illustrated in Figure 4B, one specific band with an apparent molecular mass of ∼80 kDa, most probably corresponding to the full-length Env precursor, was observed. All envelope proteins were found to be equally expressed and at the expected location on the plasma membrane. It can therefore be concluded that the lack of fusogenicity of the human, tamarin and saki EnvV2s is not simply related to a lack of expression or a mislocalization of the EnvV2 proteins.

**Figure 5:**
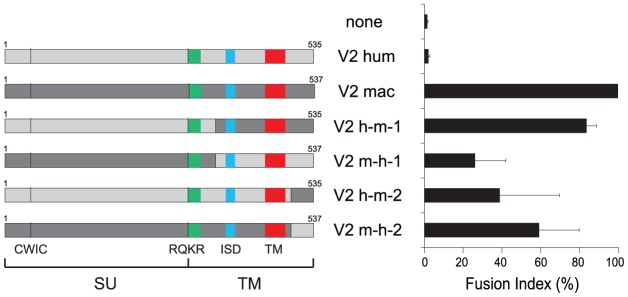
Cell–cell fusion assay for chimeric EnvV2 proteins. Structure of the chimeric envelope proteins between the human (light gray) and macaque (dark gray) EnvV2 proteins, with the characteristic Env domains indicated (same color code as in Figure 2 for the fusion peptide, the immunosuppressive domain (ISD) and the transmembrane domain (TM), and the positions of the CWIC motif and the SU/TM cleavage site RQKR). 293T cells were transfected with the chimeric EnvV2-expressing plasmids, stained 48 h after transfection with May-Grünwald-Giemsa, and fusion indexes were measured as in Figure 3.

A second series of experiments was further devised to determine whether the differences in fusogenic activity among the EnvV2 proteins could be due to a shift in or loss of their capacity to recognize their cognate receptor. Accordingly a series of chimaeras were constructed and assayed for fusion activity. As illustrated in Figure 5 for the human/macaque chimaera, the loss of function of human EnvV2 is not simply due to a defect in the SU moiety –which carries the receptor binding domain- since the human SU is fully active when associated with the macaque TM subunit. Yet the reverse chimaera retains some fusion activity, suggesting that the human TM cannot be considered as defective *per se*, but rather that fusogenicity is the result of complex structural and dynamic interactions between both subunits, as examplified for several retroviral envelope proteins by specific mutations/truncations (reviewed in [31]). This conclusion is further supported by the two reverse chimaeras involving only the exchange of the cytoplasmic tails between the human and macaque proteins, which both retain some fusion activity.

In conclusion, the *envV2* gene has clearly conserved, in all the tested Old World monkeys, the fusogenic activity that was most probably associated with the ERV that primitively entered the primate lineage, but this property has been lost on several occasions, including in the human lineage, in some New World monkeys, and to some extent in non-human hominoids. It can be also concluded that this loss of fusion activity is not associated –at least for the human gene- with an inadequacy between the receptor for this protein and the EnvV2 SU subunit. This raises two questions: first, can *envV2* still be a *“syncytin”* in Old World monkeys, and second what can be the function –if any-, of this conserved gene in the other primates.

### Conservation of the placenta-specific expression of *envV2* at the materno-fetal interface in the macaque Old World monkey

**Figure 6:**
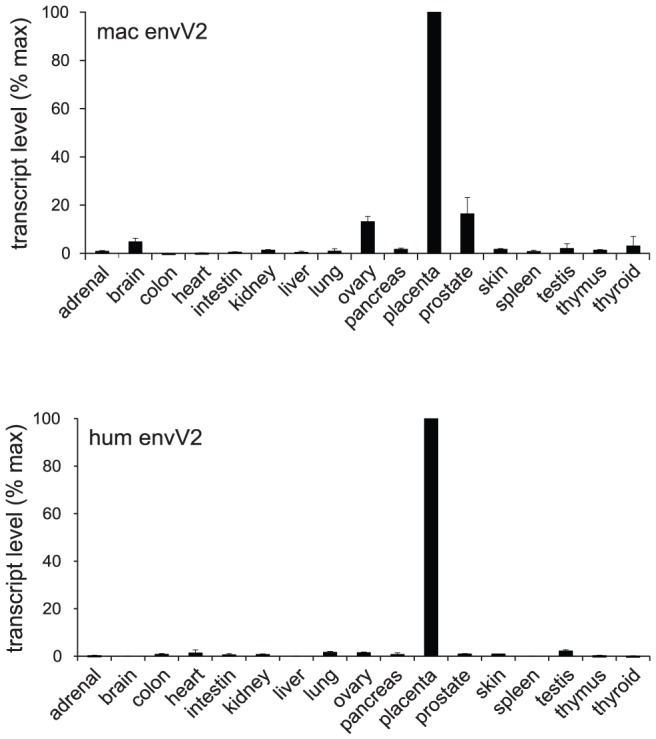
qRT–PCR analysis of *envV2* transcripts in macaque and human tissues. Real-time qRT-PCR analysis of the macaque and human *envV2* transcripts in a panel of 17 macaque (upper) and human (lower) tissues, with transcript expressed as percent of maximum, after normalization with a control gene (PPIA) mRNA (see Methods).

The above fusion assays together with the conservation of the gene for million years of primate evolution strongly suggest that *envV2* could be a *syncytin* gene, still active in the Old World monkey lineage. To test this hypothesis, the third canonical characteristic feature of *syncytins* was investigated, namely the restriction of its expression to the placenta, at the level of the specific cytotrophoblast/syncytiotrophoblast cells which contribute to the formation of the materno-fetal interface (reviewed in [1], [2]). First, as illustrated in Figure 6, a quantitative RT-PCR for *envV2* expression in a large panel of macaque tissues –including the placenta- clearly demonstrated placenta-specific expression, which was found to be at least 10-fold higher than in any other tissue tested. The pattern of expression of *envV2* in the macaque is closely related to that of the orthologous gene in humans (Figure 6).

**Figure 7:**
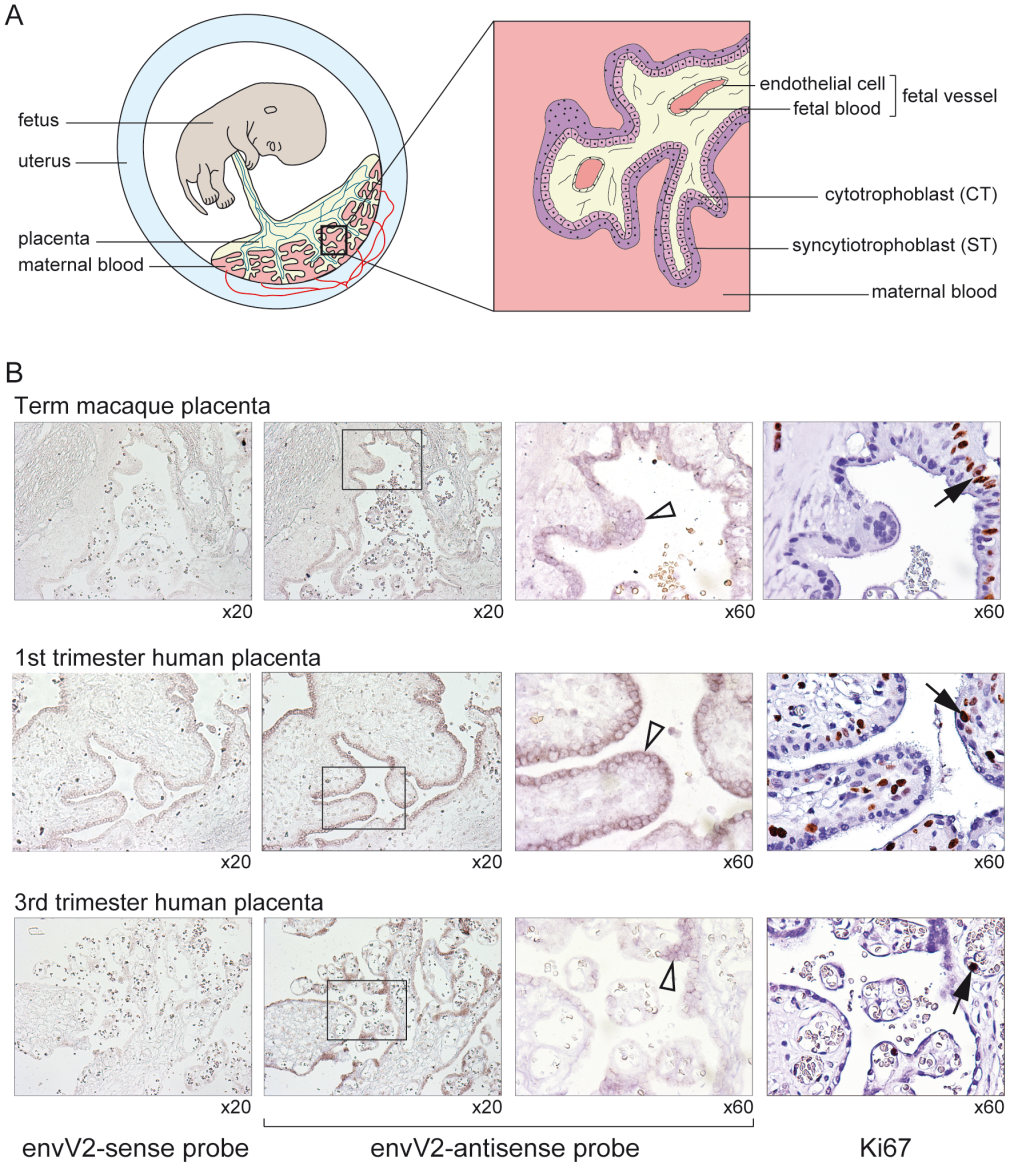
*In situ* hybridization for *envV2* expression in the macaque and human placenta. (A) Schematic representation of the simian placenta, with an enlarged villus bathed in maternal blood and displaying –from the maternal to the fetal side- the syncytiotrophoblast (ST) layer, the mononucleated cytotrophoblasts (CT) and the fetal vessels. (B) Serial sections of macaque (upper row) and human (two lower rows) placenta. (columns 1–3) *in situ* hybridization with digoxigenin-labeled *envV2* sense (negative control, column 1) and antisense (columns 2,3) riboprobes, revealed with an alkaline phosphatase-conjugated anti-digoxigenin antibody. (column 4) immunohistochemical staining of the Ki67 nuclear antigen. (columns 3,4) enlarged views with the empty arrowheads pointing to the syncytiotrophoblast positively stained for *envV2* mRNA, and the filled arrows to Ki67-marked mononucleated cytotrophoblasts.

The placenta of simians is of the hemochorial type and is characterized by the presence of fetal villi in direct contact with maternal blood [32]–[35]. The villi have an inner mononucleated cytotrophoblast layer underlying the surface syncytial layer, the syncytiotrophoblast (Figure 7A). During gestation, the cytotrophoblast layer becomes discontinuous, whereas the syncytiotrophoblast remains continuous although it can develop regions of unequal thickness, with accumulation of nuclei in some areas. The macaque and human placentae are closely related, notably at the level of the structure of the placental villi [36]–[39]. To localize precisely *envV2* expression, *in situ* hybridization on macaque placental serial sections was performed with specific digoxigenin-labeled antisense probes as well as with the corresponding sense probes as controls. As illustrated in Figure 7B, specific labeling was obtained with the antisense probe, and not with the control probes. In the macaque placenta, *envV2* expression is localized at the level of the syncytiotrophoblast, at the materno-fetal interface, as expected for a gene involved in syncytiotrophoblast morphogenesis. Of note, *envV2* expression is not detected at the level of the cytotrophoblasts which can be distinguished by their mitotic activity using an anti-Ki67 antibody [40] (Figure 7B, right panels). Finally, the figure also shows a similar localization of *envV2* transcripts in the human placenta.

In conclusion, *envV2* in the macaque Old World monkey possesses the expected features for a *syncytin*, and as such could now be named “*mac-syncytin-3*” to refer to the species and to indicate that it belongs to a family clearly distinct from the *syncytin-1* and *syncytin-2* families.

### Purifying selection and conservation of *envV2* in simians: No evidence for an Old World monkey specificity

**Figure 8:**
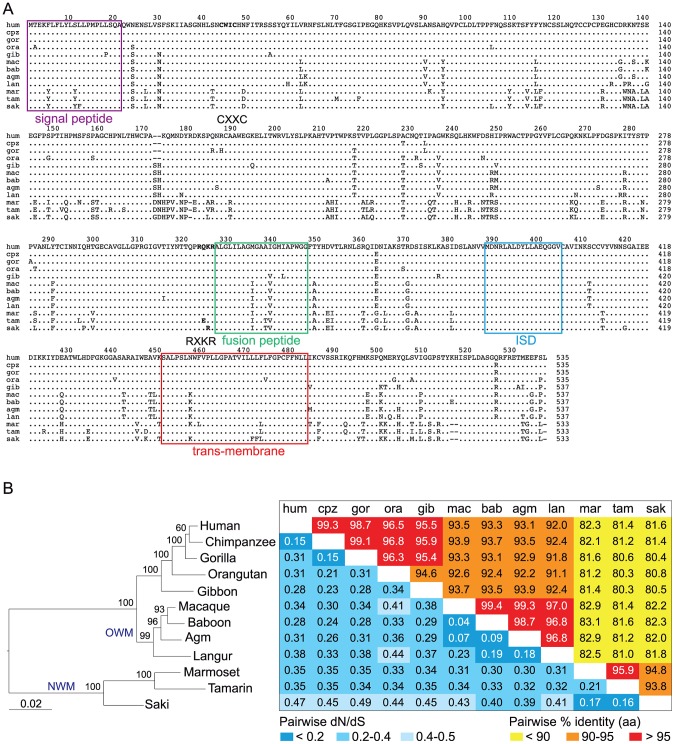
Sequence conservation and purifying selection of the *envV2* gene in primates. (A) Aligned amino acid sequences and characteristic structural features of the primate EnvV2 proteins. The SU (surface) and TM (transmembrane) subunits are delineated with the position of the putative proteolytic cleavage site (RQKR, in bold) between the two subunits, of the signal peptide (in purple) and of the CWIC motif (in bold) in the SU subunit, and the position of the fusion peptide (green), of the immunosuppressive domain (blue) and of the transmembrane domain (red) in the TM subunit. Dots indicate amino acid identity and hyphens codon deletions. Abbreviations are as in Figure 3. (B) *EnvV2*–based maximum likelihood phylogenetic tree was determined using nucleotide alignment of the *env* gene, inferred with the RAxML program. The horizontal branch length and scale indicate the percentage of nucleotide substitutions. Percent bootstrap values obtained from 1.000 replicates are indicated at the nodes. Double-entry table for the pairwise percentage of amino acid sequence identity (upper triangle) and the pairwise value of dN/dS (lower triangle) between the *envV2* genes among primate species. A color code is provided for both series of values.

As illustrated in Figure 8A, 8B, the *envV2* gene is conserved among simians: comparison of the aa sequences from the 12 genes cloned and assayed for the fusogenicity of the corresponding proteins displays high sequence conservation ranging from 80 to 99%, as well as evidence for purifying selection for the entire *env* sequences, with low values for the non-synonymous to synonymous substitution ratios (dN/dS) between all pairs of species (methods in [41]), ranging from 0.04 to 0.49 (Figure 8B). This pattern of dN/dS ratio is classically observed for cellular genes with a physiological function, in which non-synonymous mutations are strongly selected against. We obtained a similar pattern for *syncytin-2* whose entry into the primate lineage coincides with that of *envV2* and that we analyzed for the same extended set of species, with dN/dS ratios ranging from 0.08 to 0.47 (not shown). Of note, a similar analysis carried out on *envV1* (after elimination of the stop/frameshift mutations for some of the sequences) provides related low dN/dS values, a paradoxical result for a pseudogene-like sequence that can be accounted for by the frequent conversion events taking place with *envV2*, as shown in [27].

**Figure 9:**
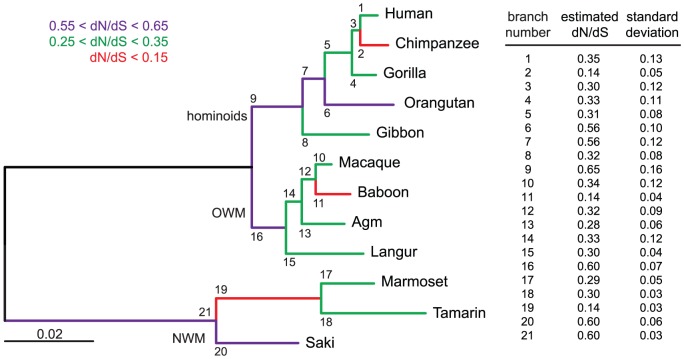
Branch-specific analysis of selection along the *envV2*-based phylogenetic tree. Analysis was performed using the GA-branch method from the HyPhy Package on the webserver www.datamonkey.org, and the selected model was the one with the best Akaike Information Criterion (AIC). Branch-specific analysis allows the non-synonymous to synonymous mutation ratio (dN/dS) to vary between phylogenetic branches. Such an analysis on the *envV2* gene reveals that all the branches are under strong purifying selection (dN/dS<1) with no significant difference in the strength of selection between New World monkeys (NWM), Old World monkeys (OWM) and hominoids. Left: maximum likelihood tree of *envV2* (same as in Figure 8) is represented together with the branch numbers. The color code for each branch class is indicated on top. Right: estimated branch-specific dN/dS values are indicated ± standard deviation.

To characterize further the conservation and evolution of the *envV2* gene and detect possible differences of selective pressure on distinct sites of the protein and/or between different branches among the gene phylogeny, we performed a more refined analysis using algorithms implemented in the PAML and HyPhy programs [42]. The site model analysis, allowing for the dN/dS ratio to vary between codons, provided support for a model (Model M7) in which most of the codons are under purifying selection (0<dN/dS<0.84, 80% of the sequence), while a few codons are evolving nearly neutrally (0.97<dN/dS<1, 20% of the sequence). The alternative model allowing for some codons to be under positive selection (dN/dS>1, Model M8) did not fit better to the data (Model M8 versus M7, degree of freedom = 2, chi2 = 1.5, p-value = 0.47). The distribution of dN/dS values is homogenous among codons, with no particular domain emerging. Analysis using the HyPhy package [43], with slightly different site-specific models (*i.e.* Random Effect Likelihood or Fixed Effect Likelihood), leads to similar conclusions. Finally, a branch model analysis using the HyPhy package (GA-Branch analysis), allowing for the dN/dS ratio to vary between branches, does not provide support for some phylogenetic branches to be under positive selection nor for significant differences in strength of selection between hominoids, Old World monkeys and New World monkeys (Figure 9). A similar analysis using the PAML package provides support for a model in which all branches are under strong purifying selection, with dN/dS = 0.36. In conclusion, no specificity for the evolution of the Old World monkey lineage can be unraveled from the sequence analyses, with the *envV2* genes from all species being under strong purifying selection.

## Discussion

The *envV2* gene entered the simian lineage >45 Mya together with *syncytin-2*, and was previously demonstrated to be conserved as a full-length envelope protein encoding gene and to be placenta-specific but not fusogenic in humans [8], [24], [27]. We show here that *envV2* has retained fusogenic activity in Old World as well as in some New World monkey species. Furthermore, in the macaque, expression of the fusogenic *envV2* gene is placenta-specific, and *in situ* hybridization demonstrates that it is expressed at the materno-fetal interface, as expected for a *syncytin* gene. Accordingly, it can be proposed that *envV2* was primitively captured and domesticated as a *syncytin* gene, prior to the simian radiation, and that its fusogenic activity was lost on several occasions, firstly before emergence of the hominoid branch, approx. 30 Mya, and then in several New World monkey species, except *Callithrix jacchus*. It shows that the fusogenic function of *envV2* is not uniformly subjected to selection, which could indicate either i) progressive degradation of this primitive *syncytin* gene, or ii) selection for another function of the encoded protein. The progressive decay of the gene could be accounted for by the presence of other *syncytin* genes captured in the course of primate evolution: *syncytin-2* is present in all simians where it has retained fusogenic activity [8], and its functions might therefore have substituted for part or all of those of *envV2* in the course of evolution; *syncytin-1* entered the primate lineage later but retained fusogenic activity in all the hominoids, whereas it was lost in Old World monkeys [5]–[7], *i.e.* in a mirror-like manner as compared to *envV2* evolution –thus consistent with a possible functional complementation between the two *env* genes. Another possibility, which would be consistent with the conservation of the full-length *envV2* ORF with low dN/dS ratios, the demonstrated branch-independent purifying selection, and the sustained placenta-specific expression, could be that fusogenic activity is not the property for which this gene has been selected for, and that there is another EnvV2 function that is being selected. We propose that this function is linked to the presence of an immunosuppressive domain (ISD) on the EnvV2 protein, a domain that we have previously unambiguously demonstrated to be functional [19]. As illustrated in Figure 8A, this sequence, which corresponds to a highly structured polypeptide domain, is the longest unaltered sequence among the 12 EnvV2 aligned sequences, with a stretch of more than 30 amino acids without a single mutation. This domain, also present in the envelope protein of infectious retroviruses and most endogenous retroviruses (with the significant exception of Syncytin-1 in primates and Syncytin-A in muroids), carries an immunosuppressive function that can inhibit both the humoral and cellular immune response, and which has been proposed to participate –among other factors- in the establishment of materno-fetal tolerance. Along this line, conservation of the canonical ISD sequence among the EnvV2 orthologs would be the driving force for the observed conservation of the EnvV2 sequence in evolution, despite the presently demonstrated variable selective pressure on its fusogenic activity. Another possible role that could have been selected for is protection against infection by the so-called process of receptor interference, with the well-known examples of the murine *Fv4 env*-like gene, and of the *enJSRV* ovine genes (reviewed in [44]). Yet, we do not favour such a role for *envV2* whose expression is severely restricted to the placenta, at variance with the *Fv4* and *enJSRV* genes which disclose a broader expression pattern as would be expected for an efficient protective effect against different routes of infection. In addition, there is no known infectious retrovirus that presently possesses an *env* gene closely related to *envV2*. Whatever the interpretation, it clearly emerges that captured *syncytins* are prone to disruption or complete deletion and, in this respect, it is interesting to note that EnvR, also an ancestrally captured retroviral *env* gene, which lost its fusogenic activity very early in the course of primate evolution (with a stop codon just upstream of the transmembrane domain of the TM subunit conserved among all simians), retained immunosuppressive activity and high-level expression in the placenta, but became dispensable, for instance in the gorilla where the entire gene is deleted, and in 1% of the human population where the gene is interrupted in a homozygous state by a premature stop codon [26], [30].

A model in which *syncytin* genes can be acquired and lost in the course of evolution is therefore very likely, and would be further consistent with the rather paradoxical situation whereby the acquisition of all the presently discovered *syncytins* has been dated between 10–80 My depending on the species, which is compatible with a fundamental role of these genes in placental mammal evolution only if one assumes that primitive *syncytins* were present earlier and have been progressively “replaced” by the stochastically acquired new *syncytins* that are presently found to be active.

## Materials and Methods

### Ethics statement

This study was carried out in strict accordance with the French and European laws and regulations regarding Animal Experimentation (Directive 86/609/EEC regarding the protection of animals used for experimental and other scientific purposes). The macaque placenta tissues used were obtained in accordance with the animal protocol approved by the Institutional Animal Care and Use Committee at the Commissariat à l'Energie Atomique (CEA). First trimester human placenta tissues were obtained from legal induced abortions and term placenta tissues after cesarean sections, with parent's written informed consent.

### Biological samples

The source of human genomic DNA is given in [45]. Chimpanzee (*Pan troglodytes*), gorilla (*Gorilla gorilla*), orangutan (*Pongo abelii*), gibbon (*Hylobates*), and cotton-top tamarin (*Saguinus oedipus*) genomic DNAs were obtained from ECACC and rhesus macaque (*Macaca mulatta*) from Zyagen. The baboon (*Papio papio*) DNA was a gift from Guy Dubreuil (Station de Primatologie, Rousset, France), the African green monkey (*Chlorocebus aethiops*) and marmoset (*Callithrix jacchus*) DNAs from Helene Gachot (Institut Pluridisciplinaire Hubert Curien, Strasbourg, France) and the Hanuman gray langur (*Semnopithecus entellus*) and white-faced saki (*Pithecia pithecia*) DNAs were extracted from blood samples provided by Baptiste Mulot (Zooparc de Beauval, Saint-Aignan, France).

Human first trimester placentas were obtained from legal induced abortions (8–12 weeks of gestation) and term placentas after cesarean section from healthy mothers near term with uncomplicated pregnancies from the Department of Obstetrics and Gynecology at the Saint-Vincent-de-Paul and Cochin Hospitals, Paris, France. Macaque term placentas were obtained from Guy Germain (CEA, Fontenay-aux-roses, France) after cesarean section on pregnant females (*Macaca cynomolgus*) at day 152 of gestation.

### Cloning of the *envV1* and *envV2* genes from simians

The orthologs of the *env* genes from the human endogenous retroviruses ERV-V1 and -V2 were PCR amplified from primate genomic DNAs. PCRs were carried out for 25 cycles (30 s at 94°C, 30 s at 56°C, and 3 min 30 s at 68°C), with sets of appropriate primers (see Table S1) in 50 µl containing 300 ng of genomic DNA, 1× AccuPrime PCR buffer II and 2 U of AccuPrime *Taq* DNA polymerase (Life technologies). *Xho*I (or *Bam*HI)-containing primers were used as forward primers and *Mlu*I-containing primers as reverse primers (Table S1). Each PCR product was then *Xho*I (or *Bam*HI)-*Mlu*I restricted and cloned into the phCMV-G vector opened with the same enzymes. Sequencing of each of the primate *env* genes was performed directly on the PCR products (Applied Biosystem sequencing kit).

For C-terminal tagging of the envelope proteins with the hemagglutinin (HA) epitope, primate *envV2*-amplified fragments were generated with appropriate primers (Table S1) from the phCMV-envV2 vectors described above. Each PCR product was restricted with *Age*I-*Xba*I and inserted in frame into a HA-containing pCMV4 plasmid (Gift of M. Malim, King's College, London, UK) opened with the *Bsp*EI-*Xba*I enzymes. The EnvV2-HA fragments were then restricted (*Sna*BI-*Eco*RV for human and macaque, *Sna*BI-*Eco*NI for marmoset and cotton-top tamarin and *Sna*BI-*Bgl*II for saki) and re-inserted into the phCMV vector.

### Cell culture

The 293T human embryonic kidney cells (ATCC CRL11268), the A23 hamster fibroblasts [46] and the G355.5 feline astrocyte cells (ATCC CRL2033) were grown in Dulbecco's modified Eagle's medium (DMEM) supplemented with 10% fetal calf serum (Life Technologies), 100 µg/ml streptomycin and 100 U/ml penicillin.

### Cell–cell fusion assays

For cell-cell fusion assays, 5×10^4^ to 1×10^5^ cells seeded in 24-well plates were transfected by using lipofectamine LTX (Life technologies) with 250 ng of *env* expression plasmid. Fusion activity of each envelope protein was visualized 24 to 48 h after transfection with the corresponding vectors by May-Grünwald and Giemsa staining, according to the manufacturer's instructions (Sigma). The fusion index, which represents the percentage of fusion events in a cell population, is defined as [(*N* – *S*)/*T* ]×100, where *N* is the number of nuclei in the syncytia, *S* is the number of syncytia, and *T* is the total number of nuclei counted.

### Cell surface biotinylation and Western blot analysis

Cell surface biotinylation assays were performed 48 h post-transfection. Cells were chilled on ice, washed twice with phosphate-buffered saline (PBS) supplemented with 0.7 mM CaCl_2_ and 0.25 mM MgSO_4_ (PBS^++^) for 30 min and then incubated with 0.75 mg/ml of sulfo-N- hydroxysuccinimide-biotin (Thermo Scientific) for 30 min on ice. Biotinylation was stopped by incubating the cells with 1 M glycine in PBS^++^ for 5 min at 4°C. The cells were washed three times with PBS/100 mM glycine, pH 7.4 and then lysed for 30 min on ice with PBS/100 mM glycine, supplemented with 1% Triton X-100 and a protease inhibitor cocktail (cOmplete ULTRA tablets, Mini Easypack, Roche). Cell lysates were incubated with streptavidin-coated magnetic beads (Dynabeads Streptavidin M-280, Life Technologies), previously washed three times with PBS/100 mM glycine, for 30 min at 4°C with gentle agitation. Biotinylated cell surface proteins immobilized on streptavidin beads were pelleted by magnetic separation and intracellular unmodified (non-biotinylated) proteins were collected from the supernatant. The streptavidin beads were then washed four times with PBS/100 mM glycine and the bead-associated proteins together with the intracellular proteins present in the supernatant were examined by Western blot analysis.

For Western blot analyses, biotinylated cell surface protein fractions were separated by SDS/PAGE on gradient precast gels (NuPAGE Novex 4–12% Bis-Tris gels, Life Technologies) and transferred onto a nitrocellulose membrane using a semi-dry transfer system. After blocking in PBS containing 0.2% Tween-20 and 5% nonfat milk, membranes were incubated 1 h at room temperature (RT) with primary antibodies, washed and then incubated with species-appropriate horseradish peroxidase (HRP)-conjugated secondary antibodies for 45 min at RT. The Super Signal West Pico Chemiluminescent substrate (Thermo Scientifics) was used to reveal proteins. HA-tagged EnvV2 proteins were detected using a rat anti-HA monoclonal antibody (3F10, Roche) and a goat HRP-conjugated anti-rat IgG secondary antibody (AbD Serotec). The antibody used to detect the cellular glyceraldehyde-3-phosphate dehydrogenase (GAPDH) was a goat polyclonal HRP-conjugated anti-GAPDH (Antibodies-online).

### Quantitative real-time RT–PCR (qRT–PCR)

Human and macaque total RNAs were extracted from frozen organs with the RNeasy RNA isolation kit (Qiagen) or obtained from Zyagen. Reverse transcription was performed with 1 µg DNase-treated RNA as in [22]. Real-time quantitative PCR was carried out with 5 µL diluted cDNA (1/25) in a final volume of 25 µL using the SYBR Green PCR Master Mix and the ABI PRISM 7000 sequence detection system (Applied Biosystems). To normalize the transcript levels, PCR amplifications using primers for the peptidylpropyl isomerase A (PPIA) gene mRNA encoding cyclophilin A were performed as an internal control.

### Histological analyses

Freshly collected placentas were fixed in 4% paraformaldehyde at 4°C and embedded in paraffin. Serial sections (7 µm) were either stained with the anti-Ki67 antibody by immunohistochemistry or used for *in situ* hybridization. For human and macaque *envV2* mRNA detection, three non-overlapping PCR-amplified *envV2* fragments of: 1) 472 bp (human) or 478 bp (macaque), 2) 324 bp and 3) 309 bp were cloned in both orientations into the pGEM-Teasy vector (Promega) to be used as templates for *in vitro* synthesis of the antisense and control sense riboprobes in the presence of T7 or SP6 RNA polymerase and digoxygenin-11-UTP (Roche Applied Science), following the manufacturer's instructions. Paraffin-embedded placenta tissue sections were processed, hybridized at 42°C overnight with each set of pooled riboprobes (human or macaque, antisense or sense) and incubated further at room temperature for 2 h with alkaline phosphatase-conjugated anti-digoxigenin antibody Fab fragments (Roche Applied Science). Staining was carried out with nitroblue tetrazolium (NBT) and 5-bromo-4-chloro-3-indolyl phosphate (BCIP) alkaline phosphate substrates, as recommended by the manufacturer (Roche Applied Science). For Ki67 immunohistochemical staining, paraffin sections were processed for heat-induced antigen retrieval, incubated with a rabbit monoclonal anti-Ki67 antibody (Neomarkers, LabVision), stained by using the peroxidase/diaminobenzidine Rabbit PowerVision kit (ImmunoVision) and counterstained with Mayer's hemalum.

### dN/dS ratio and phylogenetic analyses

Multiple alignments of sequences were carried out by using the Seaview program [47] under ClustalW protocol. Maximum likelihood trees were constructed with RAxML 7.3.2 [48] with bootstrap percentage (BP) computed after 1000 replicates, using the global time reversible (GTR) model with gamma distribution (GTR + GAMMA) and the rapid bootstrapping algorithm.

The phylogenetic analysis by maximum likelihood (PAML4) package [42] was used to run site-specific or branch-specific selection tests, and to obtain the non-synonymous vs synonymous (dN/dS) substitution rate ratios. We used likelihood ratio tests to compare the improvement in likelihood between the different models.

The site-specific and branch-specific models analyzed assumed no molecular clock (clock = 0). Site-specific models take into account a single dN/dS for all tree branches (model = 0) and a beta distribution of codons among site classes (models M7 and M8; NS site = 7 8). Branch-specific models take into account a single dN/dS for all codons within a phylogenetic group (NS site = 0), and different dN/dS values for pre-defined phylogenetic groups (model = 2). Each analysis ran until convergence (Small_Diff = 0.5e-6), and the control file is available on request. The HyPhy software package [43] was implemented on the datamonkey webserver (www.datamonkey.org) for selection analysis using two different selection tests: i) the site-specific Random Effect Likelihood (REL) and Fixed Effect Likelihood (FEL) tests, and ii) branch-specific genetic algorithm (GA)-branch analyses. The best branch-specific model was selected using the Akaike Information Criterion.

## Supporting Information

Table S1 List of primers used for amplification of genomic *envV1* or *envV2* from primates, for quantitative RT-PCR of human or macaque *envV2*, for probe synthesis used for *in situ* hybridization, and for construction of HA-tagged EnvV2 proteins.
(DOC)
