# Supplementary material for: Differential Evolutionary Fate of an Ancestral Primate Endogenous Retrovirus Envelope Gene, the EnvV Syncytin, Captured for a Function in Placentation
Source: PLoS Genet. 2013 Mar 28;9(3):e1003400. doi: 10.1371/journal.pgen.1003400 (PMC3610889; doi:10.1371/journal.pgen.1003400)
Supplement: Table S1 — List of primers used for amplification of genomic envV1 or envV2 from primates, for quantitative RT-PCR of human or macaque envV2, for probe synthesis used for in situ hybridization, and for construction of HA-tagged EnvV2 proteins. (DOC) [file pgen.1003400.s001.doc]

**Table S1**

| Primer names | Primer sequences |
| --- | --- |
| **Cloning of envV1 or V2 in Hominoidea and OWM** | |
| - Forward primer : *Xho*I-V1V2-S1 | 5’ – atcacctcgagTCAAGTGAAAAGATAAGTAAAGCCTTCTC |
| - Reverse primer EnvV1 : *Mlu*I-V1-AS4 | 5’ – atcacacgcgtGTCTGGCTACCTGCCTACCCTAA |
| - Reverse primer EnvV2 : *Mlu*I-V2-AS2 | 5’ – atcacacgcgtCTAGTGCCTT(T/G)GTTTTTATGGGAGC |
| **Cloning of envV1 or V2 in NWM** | |
| - Forward primer :  *Bam*HI-V1V2-S2m | 5’ – ctgcaggatccTGGCATTGGCTATTCCCCTCATTT |
| - Reverse primer EnvV1 : *Mlu*I-V1-AS5m | 5’ – atcacacgcgtGCCTACCTAACTGCCTACCCAAA |
| - Reverse primer EnvV2 : *Mlu*I-V2-AS4m | 5’ – atcacacgcgtGTGCCTGAGGAGATTTTATGGGAGC |
| **qRT-PCR** | |
| - Forward primer : Hum-envV2-F | 5’ – CATGACTTTGGAAAAGGAGG |
| - Reverse primer : Hum-envV2-R | 5’ – GCCAAAGAGGAAAAGTAAGAGT |
| - Forward primer : Mac-envV2-F | 5’ – CATGACTTTGGAAAAGGAGG |
| - Reverse primer : Mac-envV2-R | 5’ – ACCAAAGAGGAAAAGTAAGAGT |
| ***In situ* hybridization probe synthesis** | |
| Probe 472/478 bp : | |
| - Forward primer : EnvV2-F5 | 5’ – CATCACCAGGTCCTCATCTTACC |
| - Reverse primer : EnvV2-R4 | 5’ – GGGACAGTGTGTGCCTTGGGAAGC |
| Probe 324 bp : | |
| - Forward primer : EnvV2-F6 | 5’ – GCCTGTACCCCTCCTGGCTATG |
| - Reverse primer : EnvV2-R5 | 5’ – TTGTCTGGAGAGATTTCTGAGGG |
| Probe 309 bp : | |
| - Forward primer : EnvV2-F | 5’ – CATGACTTTGGAAAAGGAG |
| - Reverse primer : EnvV2-R6 | 5’ – AAATTCCTCCATAGTTTCCCGG |
| **EnvV2-HA fusion at the C-term** | |
| - Forward primer : Hum-ATG*-AgeI*-S | 5’ – atcaccggtcATGACAGAGAAATTCCTTTTCC |
| - Reverse primer : Hum-HA-*XbaI*-AS | 5’ – atatctagaGAGAGAAAATTCCTCCATAG |
| - Forward primer : Mac-ATG*-AgeI*-S | 5’ – atcaccggtcATGACAGAGAAATTCCTTTTCC |
| - Reverse primer : Mac-HA-*XbaI*-AS | 5’ – atatctagaGAGAGGAAATCCCTCCATAG |
| - Forward primer : Nwm-ATG-*AgeI*-S | 5’ – atcaccggtcATGACAGAGAAATACCTTTTCC |
| - Reverse primer : Nwm-HA-*XbaI-*AS | 5’ – atatctagaAAGAAACTCCCC(A/C)GT |
